# DianaHealth.com, an On-Line Database Containing Appraisals of the Clinical Value and Appropriateness of Healthcare Interventions: Database Development and Retrospective Analysis

**DOI:** 10.1371/journal.pone.0147943

**Published:** 2016-02-03

**Authors:** Xavier Bonfill, Dimelza Osorio, Ivan Solà, Jose Ignacio Pijoan, Valentina Balasso, Maria Jesús Quintana, Teresa Puig, Ignasi Bolibar, Gerard Urrútia, Javier Zamora, José Ignacio Emparanza, Agustín Gómez de la Cámara, Ignacio Ferreira-González

**Affiliations:** 1 Clinical Epidemiology and Public Health, Institute of Biomedical Research (IIB Sant Pau)-Iberoamerican Cochrane Centre, Barcelona, Spain; 2 CIBER de Epidemiología y Salud Pública (CIBERESP), Madrid, Spain; 3 Facultat de Medicina, Universitat Autònoma de Barcelona, Barcelona, Spain; 4 Facultad de Ciencias de la Salud Eugenio Espejo, Universidad Tecnológica Equinoccial, Quito, Ecuador; 5 Unidad de Epidemiología Clínica y Soporte Metodológico, UICEC de BioCruces-SCReN, Barakaldo, Spain; 6 Unidad de Bioestadística Clínica, Hospital Universitario Ramón y Cajal, IRYCIS, Madrid, Spain; 7 Unidad de Epidemiología Clínica, Hospital Universitario Donostia, BioDonostia, San Sebastian, Spain; 8 Unidad de Investigación Clínica, Hospital Universitario 12 de Octubre, Madrid, Spain; 9 Departmento de Cardiología, Hospital Universitari Vall d'Hebron, Barcelona, Spain; 10 Vall d'Hebron Institut de Recerca (VHIR), Barcelona, Spain; Renal Division, Peking University First Hospital, CHINA

## Abstract

**Objective:**

To describe the development of a novel on-line database aimed to serve as a source of information concerning healthcare interventions appraised for their clinical value and appropriateness by several initiatives worldwide, and to present a retrospective analysis of the appraisals already included in the database.

**Methods and Findings:**

Database development and a retrospective analysis. The database DianaHealth.com is already on-line and it is regularly updated, independent, open access and available in English and Spanish. Initiatives are identified in medical news, in article references, and by contacting experts in the field. We include appraisals in the form of clinical recommendations, expert analyses, conclusions from systematic reviews, and original research that label any health care intervention as low-value or inappropriate. We obtain the information necessary to classify the appraisals according to type of intervention, specialties involved, publication year, authoring initiative, and key words. The database is accessible through a search engine which retrieves a list of appraisals and a link to the website where they were published. DianaHealth.com also provides a brief description of the initiatives and a section where users can report new appraisals or suggest new initiatives. From January 2014 to July 2015, the on-line database included 2940 appraisals from 22 initiatives: eleven campaigns gathering clinical recommendations from scientific societies, five sets of conclusions from literature review, three sets of recommendations from guidelines, two collections of articles on low clinical value in medical journals, and an initiative of our own.

**Conclusions:**

We have developed an open access on-line database of appraisals about healthcare interventions considered of low clinical value or inappropriate. DianaHealth.com could help physicians and other stakeholders make better decisions concerning patient care and healthcare systems sustainability. Future efforts should be focused on assessing the impact of these appraisals in the clinical practice.

## Introduction

Healthcare systems worldwide must promote the most effective interventions and avoid those that are of low-value or inappropriate in order to face the challenge of remaining sustainable without jeopardising the quality of care [1–3]. Assessing appropriateness in health care involves three dimensions: 1. effectiveness, including the risk-benefit trade-off based on valid evidence; 2. cost-effectiveness, taking into account the available resources, and 3. characteristics, values and preferences of the individual, the community and society [4,5]. In recent years, the concept of value in healthcare, defined as outcomes relative to costs, has been introduced to better reflect whether a medical procedure is justified in the face of its benefits and costs [6]. Other authors have preferred the terms overuse or underuse to describe inappropriate interventions [7].

Using one term or another and applying a variety of methods, several researchers and clinical experts around the world have assessed or given their opinion about the appropriateness or the value of many healthcare interventions. Over the last years, a number of initiatives have been established to address this topic [8–14]. Information about these initiatives and their appraisals has been disseminated through different formats, such as research articles, letters, institutional reports and websites. Furthermore, the information is widely dispersed, making it difficult and inefficient for any potential user, either caregivers, policy makers, or patients, to form a complete view of what has been published on this topic. To solve these problems and to disseminate these initiatives and their results as widely as possible, we developed an on-line database that could serve as a fast, user-friendly, and constantly updated source of information concerning healthcare interventions appraised for their clinical value and appropriateness. In this article we describe the process of building the on-line database and we present the features of the website where it is hosted and the results of a retrospective analysis about the initiatives and the appraisals that were included in the website until July 2015.

## Materials and Methods

### Database development

In order to develop the database we established the following definitions:

- Appraisal: any assessment or critical judgment about any healthcare intervention considered either as low-value, inappropriate or unnecessary, or valuable but underused, in the form of a clinical recommendation, literature review or an expert’s analysis.
- Healthcare intervention: any treatment (e.g. drug, surgery, procedure, therapy or counselling), any test (e.g. laboratory, imaging, or any diagnostic procedure) or any other action (e.g. educational or management strategy) used in any field of healthcare to improve health or to help with a particular problem.
- Initiative: any collaborative effort to either appraise the appropriateness or the value of healthcare interventions or to collect clinical value and appropriateness appraisals.

The development of the on-line database started in Jun 2012. First, we searched the initiatives worldwide assessing the appropriateness or the value of healthcare interventions and their appraisals. We then defined and obtained the information necessary to build the database. Finally we designed the website where the database would be hosted, including a search engine to consult the database. The process to create the database ended in January 2014 with the launching of the website.

#### Search strategy and selection of references

**Fig 1:**
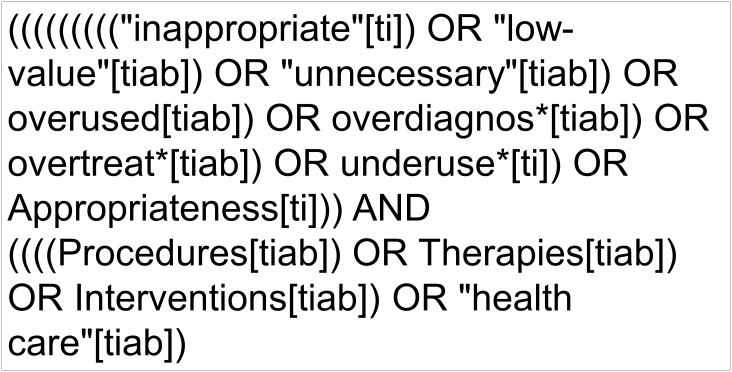
Search strategy to identify initiatives.

We searched the main worldwide initiatives that aimed to assess the appropriateness or the value of healthcare interventions and their appraisals by following medical news and article references, and by contacting experts in the field. We also searched articles related to the initiatives in MEDLINE (PubMed) using the strategy shown in Fig 1. The search was limited to articles published after June 2008 but no language restrictions were applied. Additionally, we used Google to search more information about the initiatives and about the authors of the appraisals.

We selected any type of publication (e.g. research article, letter, review, etc) as long as it met all the following criteria:

- The reference either contains a set of appraisals about low-value, inappropriate or unnecessary interventions or it is part of an established initiative according to the definition provided above.
- The authors of the appraisal and of the initiative, if they are different, must belong to non-profit research or academic groups, such as scientific societies, or to governmental agencies or institutions.

We also searched for other appraisals published by the initiative but not included in the publication found with the search strategy.

#### Data extraction and database building

A team of trained physicians retrieved the following information regarding the initiatives: institutions involved, country, year of launching, funding, aim of the initiative and methodology used to make the appraisal. The team also obtained the following information regarding the appraisals: initiative, publication year, type of intervention (e.g. diagnostic, pharmacologic or preventive), related specialties (e.g. anaesthesiology or cardiology), recommended action regarding the intervention (i.e. in favour or against its use), and keywords identified in the title. When appraisals were in languages other than English or Spanish and the titles clearly stated the population and the intervention, the team translated the title directly into English and Spanish with the support of a translator. If titles were not clear, they added a short explanation in English and Spanish.

The database was created after identifying, selecting, classifying and translating the appraisals.

#### Website design

We designed and created the website with the help of a team of IT engineers. The design included a search engine to retrieve the appraisals from the database and other content such as a news section, a list of the authoring initiatives, and a section where users could suggest new content for the website.

The web app was developed in Hypertext Pre-processor (PHP) 5. We used the latest technologies in web development, such as Hyper Text Markup Language (HTML5), cascading style sheets (CSS3) and Asynchronous JavaScript-XML (Ajax). We also used MySQL 5 as a database management system in order to perform query optimization. The application was installed on a Linux server with redundant elements to ensure stability, and Secure Sockets Layer (SSL security).

### Retrospective analysis

We analysed the initiatives and the appraisals included in the database until July 2015. Data for this analysis were obtained directly from the website. We described the following characteristics of the initiatives: researchers and institutions involved, country, year of launching or publication, type of initiative, terms used to describe their aims, and number and type of appraisals.

We analysed the appraisals included in the database according to the following characteristics: specialty, type of intervention concerned in the recommendation, and publication year. We also analysed the appraisals that had been published on selected topics and some findings of the database maintenance process.

## Results

### Features of the on-line database

**Fig 2:**
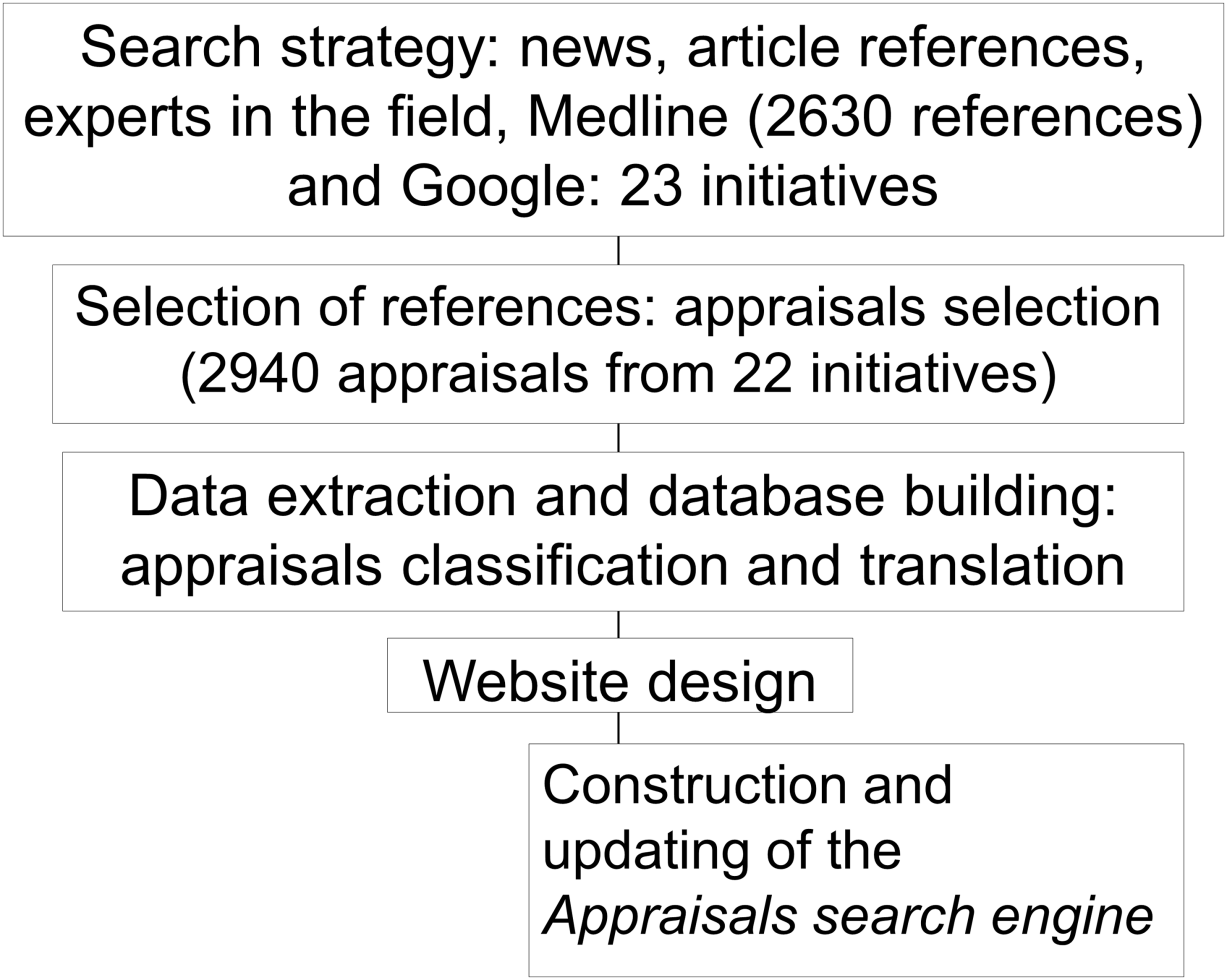
Process to create the on-line database www.DianaHealth.com.

Fig 2 summarises the database development.

The on-line database is hosted at www.DianaHealth.com in English, and also at www.DianaSalud.com in Spanish. The name of the website is an acronym that stands for its objective: Dissemination of Initiatives to Analyse Appropriateness in Healthcare (Divulgación de Iniciativas para Analizar la Adecuación en Salud). The database and the website where it is hosted are open access; they meet the Open Source Initiative criteria.

The website has seven sections: *Initiatives*, *Search recommendations/analysis*, *News*, *Report a new recommendation or suggest a new analysis*, *Contact us*, *About us*, *and How to search*.

The section *Initiatives* comprises a brief profile of each initiative which includes the following information: authors, year of launching, aim, and a link to its respective website.

In the section *Search Recommendations/Analysis*, users can consult the appraisals database through a search engine. The engine admits one or more of the following criteria: initiative, medical specialty, type of intervention, type of appraisal (high value or low value) or publication year. The search engine also admits specific free-text search terms related to any health problem or topic, such as, aortic aneurysm, epilepsy, bevacizumab, or vaccines. The search terms can be entered in either English or Spanish. After running a search, the list of results is displayed on the right. Each item in the list is an appraisal of the value or appropriateness of a given healthcare intervention. By clicking on any result, users can find additional information such as the authoring initiative, title, publication year, specialties involved, type of intervention, and a link to the original source where it was published. Furthermore, the search results can be exported into an Excel spreadsheet (*.csv) containing all this information.

The *news* section announces new initiatives and appraisals included in the website. It also posts events such as conferences and meetings on topics of interest, such as overdiagnosis, right care and clinical value.

In the section *Report a new recommendation or suggest a new analysis*, users can suggest the inclusion of new initiatives or appraisals not yet included in the database. In the section *Contact us*, users can find our e-mails to suggest improvements to the website and collaborative proposals. In the sections *About us*, *and How to search* we provide information about the operation of the website. Moreover, the website has *Facebook* and *Twitter* accounts to increase dissemination of the included initiatives and their appraisals.

The website is financially independent and not for profit. It does not receive financial support of any kind from the pharmaceutical or technology industries. It has been partially funded by the CIBERESP, a governmental research consortium in Spain (see the Funding section at the end of the article).

To keep the website updated, we identify new initiatives applying the same search strategy described in the methods, every one or two months. To keep the database valid, we check the sources where the included initiatives and the appraisals were identified every month or two, looking for modifications in the appraisals, such as withdrawals. Finally, to ensure that the links to the original sources are always functional, our IT engineers developed a system that automatically detects broken or misleading links to the original sources. The system generates a weekly report with the dysfunctional links, so we can fix them almost as soon as they change.

### Retrospective analysis

Since its launching in January 2014 until July 2015, we identified 23 initiatives (Tables 1, 2 and 3) and included 2940 appraisals of healthcare interventions from 22 initiatives in the database. The appraisals from one initiative (the ACR Appropriateness Criteria [14]) have not yet been included in the database. None of the 22 initiatives included until July 2015 was identified through the option “Report a new recommendation”.

**Table 1:** Characteristics of the initiatives aimed at reducing low-value or inappropriate healthcare interventions included in DianaHealth.com until July 2015. Results are shown in order of year of launching.

Initiative (year of launching/publication)	Authors (Country)	Type of initiative	Results
1. ACR Appropriateness Criteria^®^ (1993)	American College of Radiology (United States)	Set of guidelines obtained from literature review and expert consensus	Appropriateness Criteria on over 208 clinical conditions [14,15]*
2. NICE Do not Do Recommendations (2009)	National Institute for Health and Clinical Excellence (NICE) (United Kingdom)	Set of recommendations from clinical guidelines	987 Clinical recommendations [8,16]
3. Cochrane Quality and Productivity topics (2010)	National Institute for Health and Clinical Excellence (NICE) (United Kingdom)	Set of conclusions from literature review (Cochrane systematic reviews)	63 Reports drawn from systematic reviews by the Cochrane Collaboration [16,17]
4. U.S. Preventive Services Task Force A and B recommendations (2010)	U.S. Preventive Services Task Force (United States)	Set of recommendations from clinical guidelines	20 Clinical recommendations rated as A [18]
5. The Canadian Task Force for Preventive Health Care (CTFPHC) Guidelines (2010)	The Canadian Task Force for Preventive Health Care (Canada)	Set of recommendations from clinical guidelines	30 Clinical recommendations [19]
6. JAMA Less is more collection (first article in the collection is from 2010)	JAMA Internal Medicine (International journal based in the US)	Collection of articles	208 Original investigations and other type of publications [20,21]
7. MAPAC Initiative (In Spanish, Mejora de la Adecuación de la Práctica Asistencial y Clínica. In English, Improvement of Appropriateness in the Clinical Practice and Healthcare (2011)	Centro de Investigación Biomédica en Red de Epidemiología y Salud Pública (CIBERESP) (Spain)	Initiative of our own. It provides clinical recommendations to avoid inappropriate and low-value interventions and to promote valuable interventions	14 Clinical recommendations
8. Elshaug, et al. Article (2012)	Researchers from the Comprehensive Management Framework (CMF) (Australia)	Conclusions from literature review	A list of over 150 potentially low-value health care practices [12]
9. Choosing Wisely^®^ (2012)	ABIM Foundation and national organizations representing medical specialists (United States)	Campaign gathering clinical recommendations from scientific societies	435 Evidence-based recommendations [9]; patient-friendly materials.

*The ACR Appropriateness criteria have not yet been included in the website.

**Table 2:** Characteristics of the initiatives aimed at reducing low-value or inappropriate healthcare interventions included in DianaHealth.com until July 2015. Results are shown in order of year of launching.

Initiative(year of launching/publication)	Authors (Country)	Type of initiative	Results
10. Doing more does not mean doing better (In Italian, Fare di più non significa fare meglio) (2012)	Slow Medicine (Italy)	Campaign gathering clinical recommendations from scientific societies	209 Clinical recommendations [22,23]; patient-friendly materials
11. Essencial (2013)	Agència d’Avaluació i Qualitat Sanitàries de Catalunya (AquAS) (Catalonia, Spain)	Campaign gathering clinical recommendations from scientific societies	37 Evidence-based recommendations [24]; patient-friendly materials
12. Compromiso por la calidad de las Sociedades Científicas (In English, Scientific Societies' Commitment for quality) (2013)	Ministerio de Sanidad, Servicios Sociales e Igualdad (Spain)	Campaign gathering clinical recommendations from scientific societies	105 Clinical recommendations [25]
13. Prasad, et al. Article (2013)	Researchers from several centres and universities (United States)	Conclusions from literature review	A list of 146 existing practices found to be no better than a lesser Therapy [13]
14. TheBMJ Too Much Medicine (first article in the collection is from 2013)	The British Medical Journal (International journal based in the UK)	Collection of articles	139 Original investigations, editorials, or analyses about unnecessary care [26]
15. Choosing Wisely Canada (2014)	Canadian Medical Association/University of Toronto and national societies (Canada)	Campaign gathering clinical recommendations from scientific societies	151 Evidence-based recommendations [10]; patient-friendly materials
16. Choosing Wisely Netherlands Campaign (2014)	Dutch Association of Medical Specialists (OMS), scientific associations and ZonMw (Netherlands)	Campaign gathering clinical recommendations from scientific societies	*Wise choices*: 25 clinical recommendations [11]; *Care evaluation*: effectiveness studies; Analyses of the variations in health services activity
17. Prescrire Pour mieux soigner, des medicaments à écarter: bilan (In English, Towards better patient care: drugs to avoid in 2015) (2014–2015)	Prescrire.org (France)	Conclusions from literature review	List of 71 drugs considered more harmful than beneficial [27,28]

**Table 3:** Characteristics of the initiatives aimed at reducing low-value or inappropriate healthcare interventions included in DianaHealth.com until July 2015. Results are shown in order of year of launching.

Initiative (year of launching/publication)	Authors (Country)	Type of initiative	Results
18. Recomendaciones No Hacer (In English, Do not Do Recommendations) (2014)	Sociedad Española de Medicina de Familia y Comunitaria semFYC (Spain)	Campaign gathering clinical recommendations from scientific societies	30 Clinical recommendations [29]
19. Morgan et al. Article (2014)	Researchers from several centres and universities (United States)	Conclusions from literature review	Review article providing conclusions on 10 overused health care interventions [30]
20. Smarter medicine (2014)	Swiss Society of General Internal Medicine (Switzerland)	Campaign gathering clinical recommendations from scientific societies	5 Clinical recommendations [31,32]
21. Recomendaciones No Hacer (In English, Do not Do Recommendations) (2014)	Sociedad Española de Radiología Médica SERAM (Spain)	Campaign gathering clinical recommendations from scientific societies	38 Clinical recommendations [33]
22. Choosing wisely Australia (2015)	Australia’s medical colleges and professional societies and facilitated by NPS MedicineWise (Australia)	Campaign gathering clinical recommendations from scientific societies	27 Clinical recommendations [34]; patient-friendly materials
23. Choosing wisely Japan (2015)	Researchers from the University of Tsukuba (Japan)	Campaign gathering clinical recommendations from scientific societies	5 Clinical recommendations [35]

The 23 initiatives were: eleven campaigns gathering clinical recommendations from scientific societies, five sets of conclusions from literature review, four sets of recommendations from guidelines, two collections of articles on low clinical value in medical journals, and an initiative of our own (Tables 1, 2 and 3). These initiatives came from scientific societies, governmental health institutions, and universities from high-income countries. All the initiatives but one were launched in the last decade, and they were all still active at the time of inclusion. Two initiatives, the Right Care Alliance from the US [36] and the Right Care programme from UK [37], were not included as such in DianaHealth.com since they do not provide appraisals of the clinical value or appropriateness of any particular intervention. However, they were included in the DianaHealth.com news section. The US Right Care initiative is an interesting network of healthcare professionals and citizens who promote avoidance of overuse in medicine through educational materials and other resources. The UK Right Care programme focuses on describing variability in clinical practice and compiling local examples of commissioning innovations.

The terms used by the included initiatives to describe their aims were varied. For example, Elshaug et al [12], Prasad et al [13], and the Spanish initiatives ‘Essencial’ [24] and ‘Compromiso por la Calidad de las Sociedades Científicas’ [25] refer to “low-value practices.” The US Choosing Wisely [9], the Canadian Choosing Wisely [10] and the Australian Choosing Wisely [34] described the interventions as “unnecessary.” Other initiatives did not use a specific term but referred to interventions that “should be discontinued or not used routinely” (Do not Do [8]), or to practices “that confer no benefit but have true risks” (Less is More [21]).

Most of the initiatives (15 out of 23) presented their appraisals as clinical recommendations (in favour or against the use of a given intervention), either evidence-based or based on expert consensus while eight initiatives did not provide recommendations: six of them provided evidence-based assessments, and two initiatives were topic collections and article series from two medical journals respectively, gathering articles of different types (original investigations, reviews, and letters) that appraise the value or appropriateness of healthcare interventions (Tables 1, 2 and 3). Four of the Choosing Wisely initiatives and Essencial, in addition to providing clinical recommendations, also developed informative materials to facilitate doctor-patient communication in order to improve appropriateness.

As for the 2940 appraisals included in the database, most of them were about low value or inappropriate interventions (96%, n = 2830). The rest (4%) were about appropriate interventions. We also included these appraisals in the website. Table 4 shows some characteristics of the appraisals included in DianaHealth.com by July 2015.

**Table 4:** Characteristics of the appraisals included in DianaHealth.com until July 2015.

Characteristic	n (%)
Specialty (n = 5334)*	
Internal Medicine	661 (12)
Family Medicine	547 (10)
Paediatrics	292 (6)
Cardiology	235 (5)
Gynaecology	241 (4)
Surgical specialties^#^	868 (16)
All the other specialties^¥^	2490 (47)
Type of intervention concerned in the recommendation n = 2940)	
Drugs (non-chemotherapy drugs)	935 (32)
Diagnostic (Images)	447 (15)
Diagnostic (Laboratory tests)	314 (11)
Diagnostic (Procedures)	217 (7)
Surgical procedures	205 (7)
Preventive interventions	126 (4)
Others	106 (4)
Other non-pharmacological therapies	105 (4)
More than one type of intervention	102 (3)
Chemotherapy drugs	75 (3)
Rehabilitation	60 (2)
Educational interventions	55 (2)
Radiotherapy	41 (1)
Small procedures	41 (1)
Management	35 (1)
Psychological interventions	31 (1)
Alternative Therapies	28 (1)
Diet and lifestyle	17 (1)
Publication year (n = 2940)	
2015	500 (17)
2014	585 (20)
2013	517 (17)
2012	1198 (41)
Before 2012	140 (5)

*Some appraisals were related to more than one specialty.

^#^Anaesthesiology, Cardiac surgery, General surgery, Maxillofacial medicine/surgery/Dentistry, Obstetrics, Otolaryngology/Head & Neck Surgery, Plastic and reconstructive surgery, Thoracic, surgery, Trauma and Orthopaedics, Urology, Vascular surgery.

^¥^ Anatomical pathology, Clinical analysis/biochemistry, Clinical Microbiology, Clinical Pharmacology, Critical care, Dermatology, Emergency, Endocrinology, Gastroenterology, Geriatric medicine/Elderly medicine, Haematology, Immunology/Allergology, Infectious diseases, Nephrology, Neurology/Neuropsychology, Nuclear medicine, Nursing, Nutrition and dietetics, Occupational and Environmental Medicine, Oncology, Ophthalmology, Palliative care, Psychiatry/Mental health, Public health, Pulmonology, Radiology, Radiotherapy, Rehabilitation, Rheumatology, and a special category several specialties.

Table 5 shows an example of the number of appraisals retrieved by the search engine using three keywords: cancer, pregnancy and heart disease. Another example of the options of the search engine is shown in the S1 Appendix. This is an Excel spreadsheet obtained when selecting Vascular Surgery in the field of medical speciality (n = 73 appraisals). The search engine also allows users to identify common or similar appraisals provided by different initiatives, and contrast them. For example, the use of images for low back pain in the absence of red flags was analysed in 22 appraisals against this practice, provided by 11 initiatives (Choosing Wisely from US, Canada, and Australia; Smarter Medicine, Doing more does not mean doing better; Do Not Do; Compromiso por la calidad de las Sociedades Científicas; Essencial; Less Is More; SemFYC recommendations; and Elshaug et al.).

**Table 5:** Example of the number of appraisals retrieved by the DianaHealth.com search engine in July 2015 (n = 2940), on three different health topics. The results are classified according to the initiative.

Initiative	Cancer*	Pregnancy^#^	Heart disease^¥^
1. NICE Do not Do Recommendations (UK)	92	60	20
2. Cochrane Quality and Productivity Topics (UK)	3	4	0
3. U.S. Preventive Services Task Force A recommendations (USA)	2	9	0
4. The Canadian Task Force for Preventive Health Care (CTFPHC) Guidelines (Canada)	7	0	0
5. JAMA Less is more collection (USA)	21	1	7
6. MAPAC Initiative (Spain)	2	0	0
7. Elshaug, et al. article (Australia)	19	2	3
8. Choosing Wisely^®^ (USA)	53	3	23
9. Doing more does not mean doing better (Fare di più non significa fare meglio) (Italy)	13	4	3
10. Essencial (Spain)	4	1	1
11. Compromiso por la calidad de las Sociedades Científicas (Spain)	9	1	4
12. Prasad, et al. article (USA)	6	2	15
13. TheBMJ Too much medicine (UK)	23	1	1
14. Choosing Wisely Canada (Canada)	15	2	2
15. Choosing Wisely Netherlands Campaign (Netherlands)	1	0	0
16. Prescrire Pour mieux soigner, des medicaments à écarter: bilan(France)	4	0	0
17. Recomendaciones No Hacer semFYC (Spain)	1	0	2
18. Morgan et al. article (USA)	1	0	0
19. Smarter medicine (Switzerland)	1	0	0
20. Recomendaciones No Hacer SERAM (Spain)	2	0	0
21. Choosing wisely Australia (Australia)	2	1	1
22. Choosing wisely Japan (Japan)	1	0	0
Total	282	91	82

* Using the search term *cancer*.

^#^Using *pregnan* as a root word to find *pregnancy* and *pregnant*.

^**¥**^Combining results of two searches (*coronary*, *infarct*).

After several updating processes, we observed that the initiatives updated their contents at different intervals. For instance, Less is More and Too Much Medicine posted new items weekly (sometimes daily), Choosing Wisely and Essencial published new recommendations every one or two months, and the Prescrire Initiative published a new report after a year [28]. Furthermore, we observed that some appraisals of some of the initiatives have been withdrawn by the authoring initiative, for instance, some appraisals from the NICE Do not Do recommendations database.

During the updating processes, we also identified new initiatives. In the last updating, we identified two Choosing Wisely-like new initiatives from UK [38], and Germany [39], but we were unable to identify any appraisal.

## Discussion

### Principal findings

We have developed a website named DianaHealth.com, an on-line database of appraisals about healthcare interventions considered low value or inappropriate in clinical practice. The website is open access, independent and constantly updated. It is available in English and Spanish and has a search engine to retrieve the appraisals using one or more search criteria.

Up to July 2015, the database included 2940 appraisals from 22 initiatives. Most of the initiatives (n = 11) were campaigns gathering clinical recommendations from scientific societies, outside the context of a clinical practice guideline document. The rest of initiatives were sets of conclusions from literature review (n = 5), sets of recommendations from clinical practice guidelines (n = 3), collections of articles on low clinical value in medical journals (n = 2), and an initiative of our own. The appraisals were mostly recommendations on pharmacological and diagnostic interventions, made by clinical experts from 22 initiatives of different kinds. Most appraisals were from scientific societies.

### Strengths and weaknesses of this project

DianaHealth.com contributes to disseminating initiatives and their results, facilitating the search for information about appropriateness in healthcare. The website is user-friendly because no registration is required and the initiatives and appraisals are accessible with a few clicks. Since its contents are available in English and in Spanish, DianaHealth.com makes the information accessible to people in many countries. Finally, the exportable format to a comma-separated values file (CVS file) allows users to make further analyses related to their interest, for example, identifying interventions that have been appraised by more than one initiative and may have more consensus regarding their low value.

We have identified the following weaknesses. First, some initiatives might not have been detected since we did not carry out a systematic search. However, the most well-known initiatives worldwide have been included and the website allows the inclusion of new initiatives at any moment. Second, new appraisals might not be available in DianaHealth.com until one or two months after they are published in their original sources. Third, the nature of the appraisals is diverse; for instance, some are evidence-based clinical recommendations, and others are a judgment of one or two experts, which might have an impact on the quality of the appraisals. DianaHealth.com provides links to the information published in a variety of sources, but the responsibility for their quality rests entirely on the authors, since we do not have the resources to assess the soundness of the appraisals included in the website. Finally, the impact assessment of DianaHealth.com is still limited. Even though we have received positive comments through the website’s contact service, its Facebook, and Twitter accounts, we do not yet have any statistics of usability, or other feedback.

### Strengths and weaknesses in relation to other studies and databases

As far as we know, the DianaHealth.com project is unique. We have not found any other databases that collect the main initiatives and appraisals about clinical value and appropriateness from recent years in a single site. Some of the websites of the initiatives included in DianaHealth.com [8,9,24] provide tools to search their appraisals, but these tools offer few options and allow somewhat limited searches.

Several of the initiatives included in DianaHealth.com have been referenced by other authors. For instance, Hurley [40] wrote an interesting article describing the Choosing Wisely-like initiatives, but did not mention other initiatives pursuing the same objective.

### Implications for clinicians and policymakers

The information collected in DianaHealth.com promotes awareness of initiatives concerning inappropriate or low-value interventions. Identifying and decreasing these interventions is crucial to improve the quality and sustainability of healthcare systems. Despite the importance of these appraisals, however, some caution is necessary before applying any of them to a particular setting because certain aspects analysed when conducting the appraisals (such as, cost or necessary resources for implementation), might differ between countries, between health systems, or over time. Moreover, patients’ values and preferences must always be taken into account.

### Future actions

The database has short and long-term objectives: to expand the contents of the database by including references containing a single appraisal, searching other databases beyond MEDLINE, and including the terms *disinvestment* and *de-implementation* in the search strategy; to increase the database audience, making its contents available in other languages other than English and Spanish; and to measure the impact of the database by implementing quantitative indicators, such as the number of visits. It would also be of interest to find effective ways to present the appraised interventions to patients and to assess the impact of the appraisals on the use of the interventions in the clinical practice. Finally, it would be useful to have a search filter in Medline and other databases to identify literature about low-value or inappropriate interventions.

## Supporting Information

S1 Appendix Excel spreadsheet obtained when selecting Vascular Surgery in the field of medical speciality (n = 73 appraisals). (XLSX)
